# Retrograde type A aortic dissection: a different evil

**DOI:** 10.1093/icvts/ivac264

**Published:** 2022-10-22

**Authors:** Ana Lopez-Marco, Benjamin Adams, Aung Ye Oo

**Affiliations:** Department of Cardiothoracic Surgery, St Bartholomew’s Hospital, London, UK; Department of Cardiothoracic Surgery, St Bartholomew’s Hospital, London, UK; Department of Cardiothoracic Surgery, St Bartholomew’s Hospital, London, UK

**Keywords:** Aortic dissection, Retrograde aortic dissection, Complications in aortic surgery, Frozen elephant trunk

## Abstract

Retrograde type A aortic dissection (RTAAD) can be spontaneous or secondary to the instrumentation of the descending and thoraco-abdominal aorta. It has anatomical differences compared to antegrade type A aortic dissection that impact the management and prognosis. Treatment is not standardized. We report our approach to spontaneous RTAAD in our institution between 2018 and 2022 (*n* = 15). The mean age was 60.1 years and 93% were male. Aortic valve, coronary arteries and supra-aortic trunks were spared by the dissection in 80% of the cases; distal extension to iliacs was common and lower limb malperfusion was present in 4 cases (27%). The ascending aorta was dilated at presentation in 60% of the cases. Emergency surgery with arch/FET replacement was offered to 11 patients (73%); 3 patients (20%) received a limited proximal aortic repair; 1 patient was treated conservatively. Overall mortality was 47% (100% for limited proximal repair and 22% for those who received arch/FET). We advocate for aggressive treatment of RTAAD excluding the primary entry tear to prevent immediate- and mid-term complications.

## INTRODUCTION

Retrograde type A aortic dissections (RTAAD) are less frequent than the antegrade type A form, where the primary entry tear is located within the root, ascending and/or arch of the aorta and the dissection propagates antegradely into the distal aorta.

In RTAAD, the primary entry tear occurs distal to the left subclavian artery (LSA) and the dissection propagates retrogradely into the ascending aorta. RTAAD can be spontaneous or iatrogenic, caused by open and endovascular aortic surgery, especially when the proximal aorta is already dilated over 40 mm [1].

Management of spontaneous RTAAD is currently not standardized with reports of successful treatment with optimized medical therapy, open surgery and/or endovascular repair available in the literature [1].

We aimed to review our experience and initial and mid-term outcomes in patients presenting with RTAAD.

## METHODS

Retrospective analysis of prospectively collected data for all patients admitted with RTAAD in our institution between January 2018 and July 2022.

Patient demographics, comorbidities, operative data and postoperative complications were recorded in our local database (Dendrite Clinical Systems Ltd, UK). Long-term survival was obtained from national census data (NHS Spine).

**Figure 1:**
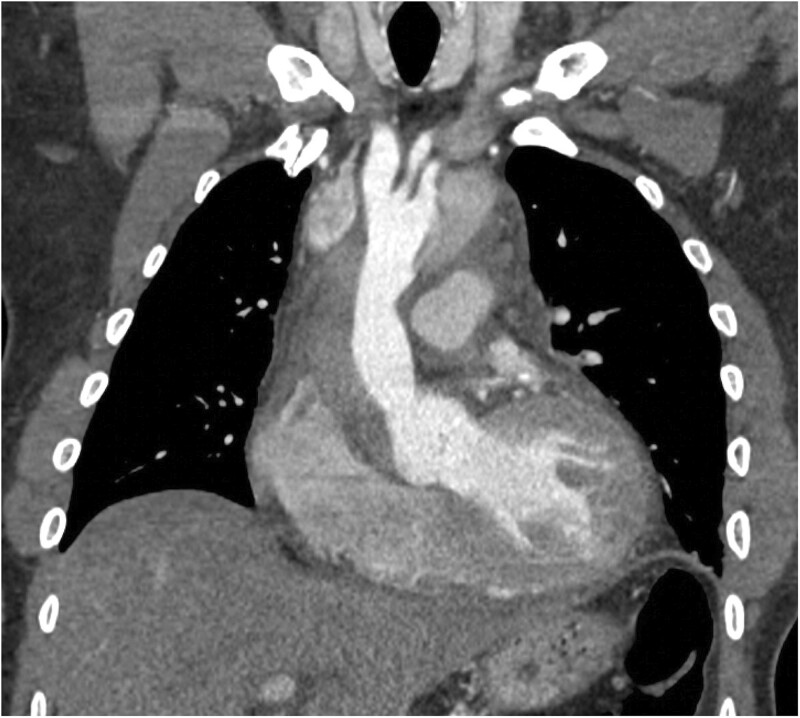
Coronal view of a contrast-enhanced CT in arterial phase demonstrating a retrograde type A aortic dissection with completely thrombosed proximal false lumen in the ascending aorta.

**Figure 2:**
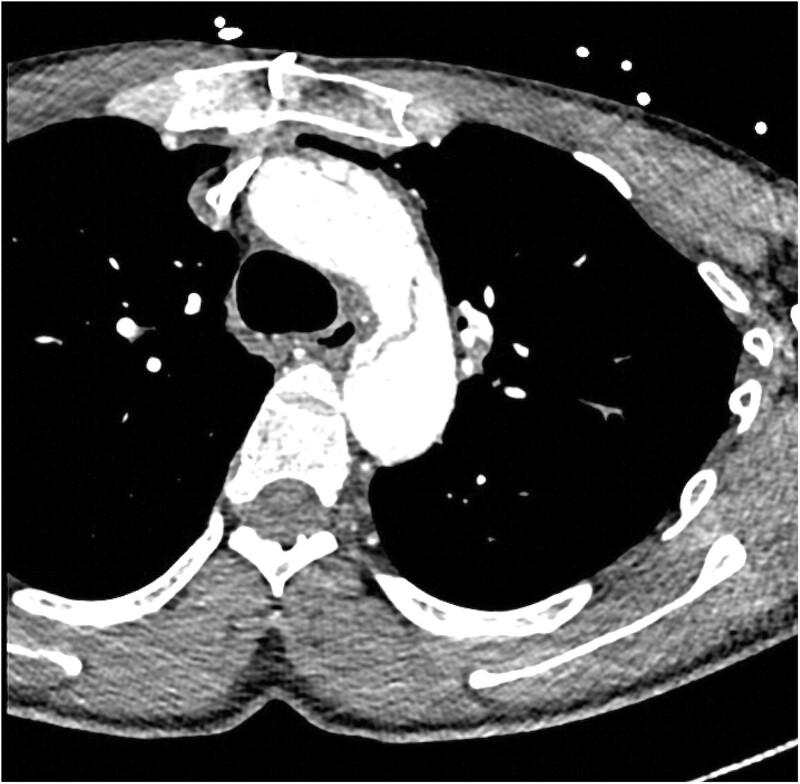
Axial view of a contrast-enhanced CT in arterial phase demonstrating a retrograde type A aortic dissection with patent proximal false lumen in the ascending aorta.

Diagnostic contrast-enhanced computed tomography (CT) scans of the aorta at presentation were analysed on Sectra PACS viewer. Aortic measurements were taken following the STORAGE guidelines [2], providing the maximal total external diameter of the aorta at the following levels: sinuses of Valsalva, ascending aorta at the level of the pulmonary bifurcation, aortic arch at the level of the left common carotid (zone 1) and proximal descending thoracic aorta (zone 3). The location of the primary entry tear and the presence of aortic dissection at each aortic level were adjudicated. Patency or thrombosis or the true and/or false lumen was determined by the presence/absence of contrast on the arterial phase (Figs 1 and 2).

Informed consent was waived by the Institutional Review Board due to the retrospective and anonymized nature of the data.

Continuous variables are expressed as mean ± standard deviation and range if normally distributed or as median and 1st and 3rd quartiles when no normal distribution. Categorical variables are expressed as percentages.

## RESULTS

Between January 2018 and July 2022, a total of 15 patients were diagnosed spontaneous RTAAD in our institution. During the same period, 266 type A aortic dissections (TAAD) were operated in our centre.

The mean age was 60.1 ± 14.2 (30 – 82) years and male sex was predominant, *n* = 14 (93%). Risk factors for aortic dissection included hypertension (100%) and Marfan syndrome, *n* = 1 (7%). One patient had valve-sparing aortic root replacement 15 years prior and another had endovascular aortic repair of a pararenal aortic aneurysm 4 months prior.

**Figure 3:**
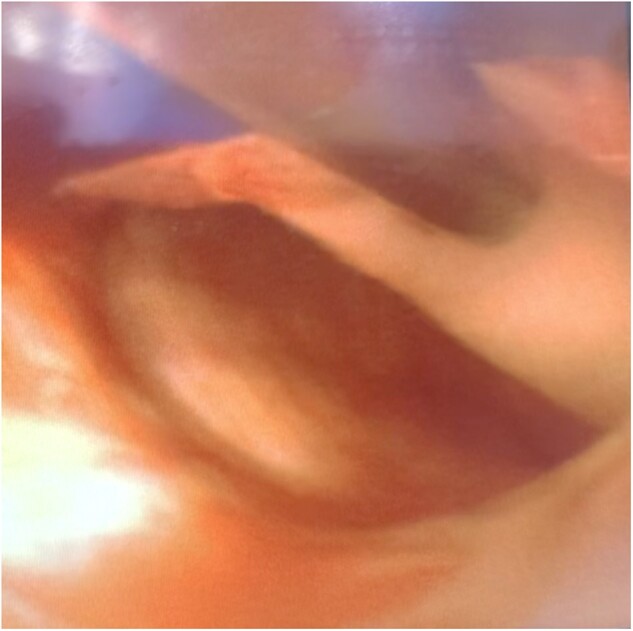
Intraoperative view of the single-use flexible bronchoscope used to inspect the arch and descending thoracic aorta during circulatory arrest. Note how the tip of the bronchoscope gently flips the dissection flap at the entry tear level.

The CT aorta revealed the presence of the primary entry tear in the proximal DTA (*n* = 12) or opposite the origin of the LSA (*n* = 2) in the junction between the lesser curve of the arch and the start of the DTA (Fig. 3). For those, with a primary tear in the proximal DTA, the tear was always identified in the segment between the origin of the LSA and the carinal bifurcation. The size of the tear varied from 0.7 to 1.2 mm on CT cross-sectional imaging.

Extent of the retrograde dissection included the aortic root in 8 cases (53%) and the ascending aorta and arch in all 15 cases as expected by the RTAAD definition. Supra-aortic trunks were affected in 3 cases (20%). The dissection extended antegradely into the TAA aorta and iliac vessels, causing acute occlusion of the left common iliac in 2 cases (Fig. 1).

The aortic valve was spared in most of the cases with mild aortic regurgitation in 4 cases, 29%. The right coronary ostia was affected by the dissection in 2 cases, mandating revascularization with a bypass graft. Only 1 patient had patent proximal FL, while the rest were thrombosed.

Aortic dimensions at presentation were as follows: aortic root 38.5 ± 3.3 mm (34–44), mid ascending aorta 42.9 ± 8.4 mm (30–70), mid arch 37.5 ± 8.5 mm (31–60) and proximal DTA 44.2 ± 9.2 mm (35–67).

Surgery was offered to 14 patients (93%) and it was performed via median sternotomy with CPB established via different cannulation strategies: ascending aorta/arch (*n* = 7), innominate artery (*n* = 3), axillary artery (*n* = 2) and/or femoral artery (*n* = 2) with venous return from the right atrium. Core temperature was cooled to 22°C and antegrade cerebral perfusion was used in all cases for cerebral protection.

Three patients had a limited aortic repair, with ascending aorta and hemiarch replacement, not being able to identify the primary tear on those locations. It was assumed that the tear noticed in the preoperative CT scan in the proximal DTA was a secondary or re-entry tear. In 2 cases, excessive bleeding from the native aorta beyond the pericardial reflection and originating at the proximal DTA was identified once cardiac ejection was reinstituted. Despite attempts to exclude the tear by extending the operation with a salvage arch and FET both patients died on table.

Eleven patients received ascending aorta and total arch/FET repair as index operation.

The remaining patient was treated conservatively due to a concomitant diagnosis of high-grade primary brain tumour with bad prognosis according to neuro-oncology multidisciplinary team.

Overall mortality for patients presenting with RTAAD was 33% (*n* = 5). Causes of death were cardiogenic shock [*n* = 2 (40%)], multiorgan failure [*n* = 1 (20%)], stroke [*n* = 1 (20%)] and exsanguination [*n* = 1 (14%)]. Those treated with a limited initial repair that did not address the initial tear had a 100% mortality, while arch/FET repair associated 13% mortality.

Other postoperative complications included stroke [*n* = 3 (20%)], spinal cord injury [*n* = 2 (13%)], tracheostomy [*n* = 7 (47%)] and haemofiltration for renal failure [*n* = 5 (33%)]. Three patients required emergency peripheral vascular surgery to treat acute lower limb ischaemia.

All hospital survivors remain alive, with a median follow-up of 0.4 years (Q1 0.31, Q3 0.94 years). Only 1 patient, with Marfan syndrome, has shown signs of distal aortic growth (4 mm in 6 weeks in the visceral aortic segment).

## DISCUSSION

Spontaneous RTAAD has been reported between 7% and 25% of all acute TAAD. Optimal treatment is not standardized with small series of different approaches available in the literature [3–8]. To exclude the primary tear during the index procedure extensive aortic replacement is required (i.e. total arch replacement with elephant trunk techniques); however, these techniques still carry higher mortality and morbidity, especially related to spinal cord injury [5, 7]. Conversely, confining the surgical repair to the proximal aorta to prevent aortic rupture causing tamponade or extension in the coronary arteries and/or aortic valve, leaves untreated the entry tear in the distal aorta, with subsequent risk of immediate or late aortic events requiring reintervention [3, 7].

Conservative medical therapy has been reported successful for cases with completely thrombosed false lumen in the ascending aorta, providing the aorta that it is not dilated beyond 55 mm [6, 8]. On the other hand, presentation with a patent false lumen in the ascending aorta, pericardial effusion, aortic regurgitation or malperfusion mandates expedited surgical treatment [3–7].

RTAAD seems to have better prognosis than the antegrade form. This might be explained by the less-frequent involvement of the aortic valve, coronary arteries and supra-aortic trunks. However, some series have reported that RTAAD associates often a more extensive distal involvement and higher presentation with malperfusion. This was observed in our series, where 3 patients required concomitant procedures to treat lower limb ischaemia.

Based on our experience, we advocate for an expedited and aggressive surgical repair with total arch/FET repair and concomitant treatment of distal malperfusion when applicable. With this approach, only 2 patients died in hospital. On the contrary, the 3 patients who underwent limited proximal aortic repair did not survive due to the acute rupture of the distal aorta near the untreated primary tear or to extensive stroke possibly contributed by intraoperative hypoperfusion.

One of the reasons for doing a limited proximal repair was the failure of recognition of the RTAAD in the preoperative CT scan. In those occasions, the presence of a large entry tear within the proximal DTA was interpreted as re-entry or secondary primary tear. Thorough interrogation of CT images, with a multidisciplinary discussion when in doubt, as well as intraoperative inspection of the entry tear, is key for a successful operation. We have recently incorporated the use of a single-use flexible bronchoscope to inspect the arch and DTA during the circulatory arrest to identify the presence of entry and re-entry tears (Fig. 3). We believe that this is especially important when there is no primary tear within the proximal aorta or the arch, as clearly indicates that the dissection has been of retrograde propagation and precludes excluding the primary entry tear within the descending thoracic aorta.

We have not observed any difference in outcomes related to the patency of the proximal FL.

There are some reports of RTAAD successfully treated with endovascular coverage of the primary tear in the DTA or using coils to induce thrombosis of the proximal FL as a bridge for definitive endovascular treatment [9, 10]. These approaches require optimal anatomy and some delays in planning that are not always permitted or recommended in TAAD.

## CONCLUSION

RTAAD has anatomical differences compared to antegrade TAAD that impact the management and prognosis. Management is yet not standardized.

We advocate for aggressive treatment for RTAAD with the exclusion of the primary entry tear to prevent immediate- and mid-term complications.

## Funding

None.

**Conflict of interest:** none declared.

## Data Availability

The data used for this manuscript it is available for review upon appropriate requests to the corresponding author.
